# Advances in Precision Therapeutics and Gene Therapy Applications for Retinal Diseases: Impact and Future Directions

**DOI:** 10.3390/genes16070847

**Published:** 2025-07-21

**Authors:** Mariam M. AlEissa, Abrar A. Alhawsawi, Raghad Alonazi, Enas Magharbil, Abeer Aljahdali, Hani B. AlBalawi, Naif M. Alali, Syed Hameed, Khaled K. Abu-Amero, Moustafa S. Magliyah

**Affiliations:** 1 Research Department, King Khaled Eye Specialist Hospital, Riyadh 11462, Saudi Arabia; mariam_al_eissa@hotmail.com (M.M.A.); syhameed@kkesh.med.sa (S.H.); kamero@kkesh.med.sa (K.K.A.-A.); 2 College of Medicine, Alfaisal University, Riyadh 11533, Saudi Arabia; raghadonazi@gmail.com; 3 Public Health Laboratory, Public Health Authority, Riyadh 12382, Saudi Arabia; 4 The Computational Sciences Department at the Centre for Genomic Medicine (CGM), King Faisal Specialist Hospital & Research Centre, Riyadh 11211, Saudi Arabia; 5 Division of Ophthalmology, Department of Surgery, College of Medicine, University of Jeddah, Jeddah 23218, Saudi Arabia; aaalhawsawi@uj.edu.sa; 6 Retina and Uveitis Department, Jeddah Eye Hospital, Jeddah 23454, Saudi Arabia; enas.mgh@hotmail.com; 7 Ophthalmology Department, King Abdulaziz University, Jeddah 21589, Saudi Arabia; aaljahdali@kau.edu.sa; 8 Division of Ophthalmology, Department of Surgery, Faculty of Medicine, University of Tabuk, Tabuk 47311, Saudi Arabia; hb.albalawi@ut.edu.sa (H.B.A.); nmalali@ut.edu.sa (N.M.A.); 9 Vitreoretinal Division, King Khaled Eye Specialist Hospital, Riyadh 11462, Saudi Arabia

**Keywords:** eyes, gene therapy, retina, gene editing, therapeutics

## Abstract

Gene therapy has emerged as a promising treatment for several eye diseases since it may restore vision and stop blindness. Many eye diseases, including retinitis pigmentosa and macular degeneration, have historically been rather difficult to treat and usually cause permanent vision loss. However, thanks to advances in gene therapy, many disorders can now be effectively targeted and genetically changed, providing a safer, more direct, maybe even curative approach. By introducing, altering, or repairing specific genes inside the eye, gene therapy seeks to fix the defective genes causing these disorders, thereby improving general eye health and visual ability. Voretigene neparvovec is one FDA- and EMA-approved treatment for *RPE65* mutations. Retinitis pigmentosa, age-related macular degeneration, X-linked retinoschisis, choroideremia, and Stargardt disease are among the several eye disorders still under clinical trials, and experimental treatment is in progress. As research on gene therapy develops, it opens the path for groundbreaking treatments that could fundamentally change the ophthalmic care scene.

## 1. Introduction

About 1 in 2000 people globally have inherited retinal diseases (IRDs). Often resulting in irreversible vision loss or blindness, these genetically inherited disorders severely tax healthcare systems and the affected individuals [1]. Currently untreatable with current medications, this group of progressive disorders includes retinitis pigmentosa (RP), Leber congenital amaurosis (LCA), and Stargardt disease. Targeting the root molecular causes of these diseases instead of only treating symptoms, gene therapy offers hope for conditions long thought to be untreatable [2]. The relative accessibility and immune-privileged environment of the eye make gene-based treatments especially appropriate for it. Approved by the FDA and EMA for RPE65-mediated LCA [3], Voretigene neparvovec (Luxturna) is the first in vivo gene therapy. Following this breakthrough treatment, the restoration of vision ability has proved both safe and efficient [4].

By means of new biotechnological approaches, including stem cell interventions, gene therapy, and nanomedicine, technological innovations and pharmacological advances are dramatically changing the therapeutic scene. By means of higher treatment efficacy, these developments could reduce care loads and improve patient outcomes [2,5,6]. Particularly, gene therapy has shown great success in treating inherited retinal diseases—clinical studies show appreciable increases in retinal function and visual acuity [2]. For some disorders, such as Leber congenital amaurosis and choroideremia, which have shown encouraging outcomes, the trend toward customized genetic therapies has been suggested [5].

Furthermore, present developments in nanomedicine help to solve issues related to traditional therapy, enhance drug distribution, and offer longer therapeutic effects [6]. These developments finally result in the development of non-invasive therapy possibilities. Notwithstanding the great promise of these innovative drugs, challenges still exist, including concerns about ethical issues, efficient delivery, and long-term safety [6]. This study will evaluate the success rates of relevant clinical trials and focus on new treatments related to gene editing technologies that have evolved in recent years.

## 2. Approved Gene Therapies for IRDs

### 2.1. Leber Congenital Amaurosis (LCA) Resulting from RPE65 Mutation

Approved by the FDA in December 2017 as the first gene replacement therapy in the United States for an inherited retinal disease, Voretigene neparvovec (Luxturna) was tracked by Daruich et al. for over 12 months to assess its effects and complications in 12 eyes of 6 pediatric patients. Best-corrected visual acuity (BCVA) showed notable improvement at the 12-month follow-up, while visual field and central macular thickness remained the same. Although high intraocular pressure (IOP) was reported postoperatively in both eyes of one patient, no intraoperative complications were noted. Crucially, except for one, all eyes showed parafoveal lamellar holes and atrophy at the injection site, and this greatly increased over the 12 months [4].

### 2.2. Retinitis Pigmentosa (RP)

Over 90 genes are currently linked to RP, a figure expected to rise with improvements in diagnostic testing [1]. Six *MERTK*-associated RP patients underwent a Phase I clinical trial in 2016 evaluating the safety and efficacy of subretinal rAAV2-VMD2-hMERTK gene replacement therapy. The rAAV2-VMD2-hMERTK construct is a recombinant adeno-associated virus serotype 2 (rAAV2) vector that carries the human *MERTK* gene (hMERTK). This gene is regulated by the VMD2 promoter, which ensures its expression specifically in retinal pigment epithelium (RPE) cells. Only one patient, though, showed ongoing visual improvement at the two-year follow-up [7]. In continuous studies, Kapetanovic et al. reported the first Phase I/II dose escalation clinical trial for X-linked RP generated by mutations in the RP GTPase regulator. An adeno-associated viral vector encoding codon-optimized human RPGR (AAV8.coRPR)-based gene replacement therapy was subretinally delivered to eighteen patients. According to the study, AAV8.coRPGR neither caused any dose-limiting side effects nor, in some patients, helped reverse visual field loss [8].

### 2.3. Choroideremia

Gene augmentation is under investigation in multi-institutional gene therapy clinical trials, which have made notable advancements in the inherited retinal degeneration brought on by *CHM* (choroideremia) gene mutations [9,10,11]. Recently published by MacLaren et al., a key Phase III clinical trial made use of the subretinal distribution of a functional *CHM* copy using an adeno-associated virus vector. The trial showed notable clinical improvements (with a two-line gain in BCVA), but it fell short of the three-line gain in BCVA [12] regulatory approval criteria.

### 2.4. Stargardt Disease

Often brought on by mutations in the *ABCA4* gene, Stargardt disease (STGD) is the most common hereditary retinal disease. For monogenic retinal diseases, gene therapy offers a possible treatment. Clinically approved adeno-associated virus (AAV) vectors do, however, have a limited loading capacity, which makes it difficult to accommodate bigger genes like *ABCA4*. Together with plasmid DNA, self-assembled nanoparticles made of (1-aminoethyl)iminobis[N-(oleoylcysteinyl-1-amino-ethyl)] propionamide (ECO), a multifunctional pH-sensitive/ionizable amino lipid, show gene transfection efficacy either equivalent to or better than the AAV2 capsid. By inducing specific and prolonged expression of ABCA4 in the photoreceptors of Abca4−/− mice, stable PEG-ECO/pGRK1-ABCA4-S/MAR nanoparticles greatly reduce the accumulation of toxic A2E in the eye. The PEG-ECO/pGRK1-102-ABCA4-S/MAR is a non-viral gene replacement approach that targets photoreceptors with the *ABCA4* gene, utilizing a GRK1 promoter and an S/MAR element to ensure prolonged expression. Several subretinal injections improve gene expression and therapeutic efficacy, reducing A2E accumulation following three doses by almost 69%. Only after several nanoparticle injections did mild inflammation show itself. Thus, PEG-ECO/pGRK1-ABCA4-S/MAR nanoparticles are a promising non-viral gene therapy for Stargardt disease type 1 (STGD1) [13].

### 2.5. X-Linked Retinoschisis (XLRS)

Ophthalmologist Josef Haas first described this disorder in 1898, noting it in two young brothers [14]. It is caused by the *RS1* mutation, though its degree and course vary greatly even among family members [15]. Treatment initiatives for this disease have historically mostly focused on lowering the schisis cavities. XLRS is a monogenic condition with a clear and rather consistent clinical phenotype; thus, it seems to be a good candidate for gene therapy [15]. Significant increases in RS1 production and secretion, as well as a decrease in cavity count, improved retinal organization, and functional rescue came from transducing RS1 into the photoreceptors of a Rs1h-deficient mouse model [16]. Because of their function in RS1 trafficking, general presence throughout the retina, and preservation even in the later stages of the disease, the transduction was extended to Müller cells in order to investigate further possible cellular targets [16]. This strategy did not reduce retinoschisis in this model, even if it produced positive results in terms of RS1 production and secretion in Müller cells [16]. This study site stresses the complexity of RS1 as a secreted protein in the extracellular space and underlines that delivering it to any retinal cell type by itself is insufficient to reduce the pathognomonic features of the disease.

### 2.6. Age-Related Macular Degeneration (AMD)

Legal blindness in people over the age of 50 in developed nations mostly results from age-related macular degeneration (AMD [17]. Approval of two intravitreal complement inhibitors—pegcetacoplan (Syfovre, Apellis Pharmaceuticals, Waltham, MA, USA) and avacincaptad pegol (Izveray, Astellas Pharma, Chuo City, Tokyo, Japan)—caused the treatment scene for geographic atrophy (GA) linked with advanced dry AMD to constantly change in 2023 [18,19]. These medications reduce the complement cascade, which is essential for the beginning of the death of retinal pigment epithelial cells and produces GA [20,21]. Many more clinical studies on wet AMD are in progress. They have shown that a therapeutic gene introduced into patient cells can routinely generate the intended protein, such as the endogenous anti-vascular endothelial growth factor (VEGF), when it integrates into them. This method offers continuous, long-term therapeutic effects, reducing the need for regular intravitreal injections.

## 3. Gene Therapy Design Issues

Although gene therapy has two main drawbacks, it is considered the most exciting treatment for RP. First, the genetic diversity and variety of mutations in RP seriously hinder the development of a generally successful treatment. Second, even in a compromised state, gene augmentation techniques for RP will only be successful if photoreceptors are still present. Therefore, achieving the best therapeutic effect depends on prompt intervention [22].

### 3.1. Modes of Delivery

While with intravitreal injection, AAV, transduces only the inner retinal cell layers, such as ganglion or Muller cells, the subretinal injection is the method of gene delivery to the outer retinal layers, as in Luxturna [23,24]. Therapeutically strong targeting of photoreceptors still depends on subretinal vector delivery, which detaches the retina and carries significant risks of collateral damage, often without reaching widespread photoreceptor transduction. Pavlou et al. defined the evolution of new engineered rAAV vectors, enabling effective targeting of photoreceptors using less invasive intravitreal administration [25].

### 3.2. Gene Replacement Against Editing

Combining the clustered regularly spaced short palindromic repeat (CRISPR) with Cas9, a new system derived from bacteria or archaea has lately been investigated as an RNA-guided DNA cleavage system. The DNA repair machinery is set off when the Cas9 nuclease cuts double-stranded DNA at a designated site, substituting the wild-type sequence for the mutant sequence [26]. Targeting several genes concurrently, made possible by the CRISPR/Cas9 method, helps to solve the problem of disease heterogeneity [27]. Already, several in vivo studies have assessed the success of this kind of treatment [28,29]. Currently, the best option for RP gene therapy is adeno-associated virus (AAV) vectors since they effectively target several retinal layers and have a great safety profile and low immunogenicity [30]. They introduce complementary cDNA, which codes for the transgene, into surviving cells. When treating a chronic condition like IR, the possibility of re-administration of AAV to the subretinal space allows long-term expression of the therapeutic gene following a single dosage, which is a desired feature [31].

## 4. The Efficacy of Gene Therapy for Eye Diseases

### 4.1. Therapy for Gene Replacement

In clinical studies on ocular gene therapy, gene replacement or augmentation therapy are the most often used methods [32]. Luxturna, a medication developed by Spark Therapeutics Inc. (Philadelphia, PA, USA), which got FDA approval for the treatment of RPE65-associated Leber congenital amaurosis (LCA), best shows the efficacy of this approach [3,33].

Luxturna delivers a functional copy of the *RPE65* gene to the retinal cells from an adeno-associated virus serotype 2 (AAV2) vector. Following suitable dilution of 1.5 × 10^11^ vector genomes in a total volume of 0.3 mL (3.35), this viral vector is injected into the subretinal space using a 41-gauge cannula via a standard pars plana vitrectomy. Usually going away in one to two days, the treatment results in a temporary subretinal bleb resembling a localized retinal detachment [3,33].

Using multi-luminance mobility testing (MLMT), which gauges a person’s capacity to traverse an obstacle course in various lighting conditions [3], Luxturna’s Phase I–III clinical studies revealed notable improvements in functional vision. Moreover, one year following therapy, there was an average improvement of 2 log units in full-field light sensitivity threshold testing (FST), compared to the control group (*p* = 0.0004 for FST; *p* = 0.0013 for MLMT). Nevertheless, measured using Humphrey visual field testing, the improvements in best-corrected visual acuity (BCVA) and macular sensitivity did not reach statistical significance [3].

Post-marketing data confirmed Luxturna’s therapeutic efficacy, especially in enhancing visual performance under low-lighting conditions [4,34,35,36,37,38,39,40]. Phase I–III trial results showed that patients can experience the noted effects up to one year and as early as thirty days post-treatment [3,33]. Studies on MLMT and FST have revealed that these advantages can even last for up to five years and seven years, respectively [41,42,43].

Safety-wise, no vector-related side effects were noted [3,33]. Among the procedure-related complications were transient and reversible ones, including cataracts, retinal tears, and increased intraocular pressure [33]. There were observed minimal immune responses, and no appreciable immunogenicity [43]. Recent complications not seen in Phase I–III trials, however, have surfaced, including chorioretinal atrophy within and beyond the bleb, inflammation, subretinal hemorrhage, subretinal neovascularization, subretinal deposits, and paracentral scotomas [36,38,39,40,42,44,45,46,47].

### 4.2. Treatments Based on CRISPR

For the treatment of several retinal diseases, the CRISPR/Cas system shows a quite promising genome-editing tool [48]. Targeting conditions including CEP290-associated Leber congenital amaurosis 10 [LCA10], rhodopsin-associated autosomal dominant retinitis pigmentosa (RHO-adRP), wet age-related macular degeneration, proliferative diabetic retinopathy, and proliferative vitreoretinopathy, this novel approach has been used [48]. Following a subretinal injection of an adeno-associated viral vector (AAV5) to provide CRISPR components to photoreceptor cells, Phase I/II clinical trials investigating a CRISPR/Cas-based therapy for LCA10 caused by *CEP290* showed notable improvements in best-corrected visual acuity (BCVA), full-field sensitivity testing (FST), visual function navigation (VFN), and vision-related quality of life (VFQ). Especially, there were no recorded negative events connected to the CRISPR/Cas intervention [49,50]. Notwithstanding these encouraging findings, there is still cause for concern about off-target effects—a phenomenon whereby unintentional genomic changes take place at areas sequentially similar to the target site [18,19]. Dealing with these risks and guaranteeing the long-term safety of CRISPR/Cas-mediated treatments in retinal diseases depends on future research.

### 4.3. The Impact of Immune Response and Cell Lifespan on the Outcome

Although clinical and preclinical studies have shown that topical, local, or systemic steroids can efficiently control inflammatory responses following ocular gene therapy, these responses are generally mild [51]. Severe inflammation can occasionally develop, though, and in these cases, quick treatment is needed to stop permanent vision loss [51]. Several elements affect the degree of inflammation and immune response, among them the kind of vector used [51]. While non-viral vectors are usually regarded as safer, viral vectors usually pose a greater risk of immunogenicity [51]. Furthermore, it is important to consider the route of administration—subretinal injections typically cause less inflammatory reactions than intravitreal injections, and suprachoroidal delivery causes even less inflammation than the intravitreal method [51]. Moreover, the dose of the viral vector influences immune activation, where higher doses correspond with sight-threatening ocular inflammation [51]. More research is required to clarify our knowledge of inflammatory reactions following ocular gene therapy and to identify the most appropriate treatment plan for these reactions.

## 5. Availability of Gene Therapy for Eye Conditions

### 5.1. Current FDA- and EMA-Approved Treatments

RPE65-associated Leber congenital amaurosis (LCA) is the only hereditary retinal dystrophy the the U.S. Food and Drug Administration (FDA) has approved for treatment at present. The treatment Voretigene neparvovec (LuxturnaTM) received approval from FDA in 2017 (https://www.fda.gov/vaccines-blood-biologics/cellular-gene-therapy-products/luxturna) (accessed on 1 April 2025) and the European Medicines Agency (EMA) in 2018 (https://www.ema.europa.eu/en/medicines/human/EPAR/luxturna) (accessed on 2 April 2025). Since then, many other nations have likewise approved this gene replacement treatment.

### 5.2. Current Clinical Trials

**Figure 1:**
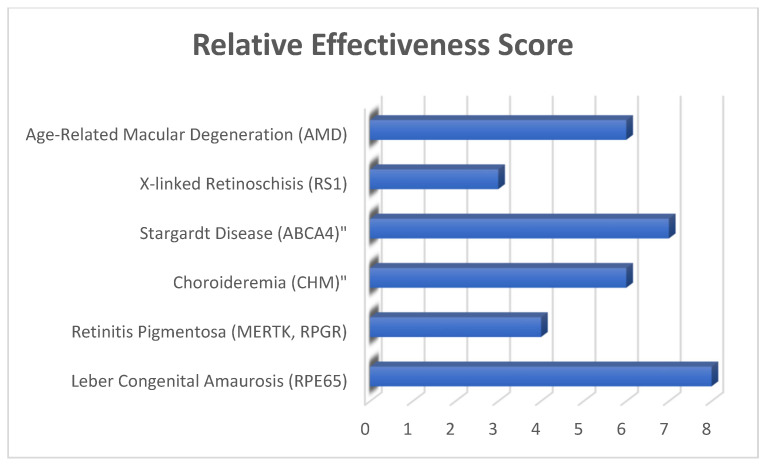
Gene therapy for major inherited retinal diseases. Specific inherited eye diseases on the Y-axis and on the X-axis relative effectiveness scores are rated from 1 to 10 depending on trial result, durability, and clinical outcomes. High score determined due to FDA approval and long-term effectiveness, and low score due to limited outcome, like therapies still in trial or preclinical stages.

Ocular gene therapy, which modulates the expression of genes in the eye, shows great potential for treating eye diseases. Though most human clinical studies are still in the research phase, they are beginning to produce interesting results. A synopsis of current retinal disease clinical studies in progress is provided in Table 1, with selected examples also detailed in Figure 1.

## 6. Trials in Gene Therapy: Difficulties

For treating particular retinal diseases, gene therapy offers a hopeful solution. Still, there are several major obstacles to including this technology in clinical practice.

### 6.1. Scarcity of Specialized Centers

One main issue is the dearth of specialized centers staffed by ophthalmic genetics experts ready to conduct necessary genetic testing and diagnostics. Determining eligibility for gene therapy or clinical trial participation depends on a molecular diagnosis. Moreover, treatments like Luxturna are limited to a small group of patients still with functioning retinal cells. This emphasizes the need for early genetic tests to find patients at the start of their disease progression, enabling them to take reasonable consideration of these treatments. Furthermore, to guarantee appropriate subretinal gene distribution, the administration of gene therapy sometimes calls for a process called pars plana vitrectomy. As it requires particular surgical knowledge and training, this greatly reduces the number of medical professionals qualified to offer gene treatments.

### 6.2. High Costs and Insurance Restraints

Cost remains a major worldwide issue as gene therapy moves from experimental research to commercial treatment. Although new technologies like CRISPR and optogenetics show promise in lowering the cost of gene therapy, Luxturna is the only treatment now accessible, costing almost USD 425,000 per eye [52,53]. Usually treated within six days, the second eye is used to lower the risk of an immune response and brings the overall cost to about USD 850,000 [52,53]. While some commercial insurers today cover gene therapy, significant gaps still exist, especially for patients enrolled in state Medicaid programs, which are government-funded medical services meant to help low-income individuals [53]. Access to gene therapy is further complicated by this financial load as well as scant knowledge of long-term safety and efficacy. Notwithstanding these obstacles, the field of gene therapy keeps developing and has the potential to revolutionize treatment for hereditary retinal diseases, thus inspiring hope that these treatments will be more generally accessible and reasonably cost-effective.

### 6.3. Regulatory and Ethical Barriers

Ocular gene therapy’s development and availability are affected by major ethical and legal issues that vary between nations as well. Ethical issues usually center on fair access since these treatments are costly and might only be accessible in wealthier countries, casting doubt on global justice [54]. Furthermore, the application of genetic modification technologies, such as CRISPR, has spurred debates on the possible side effects, especially when gene editing changes germline cells and results in heritable modifications [21]. The contentious case of Chinese scientist He Jiankui, who claimed to have produced the first gene-edited children, brought ethical questions and the dangers of pushing forward without appropriate control [55]. China has responded with rigorous laws, including criminal penalties for unapproved gene editing [56]. Moreover, different countries have very different regulatory systems, which result in different approval procedures. For instance, other nations might lack the knowledge required to properly assess these cutting-edge treatments, even while the U.S., Europe, and Japan have set clear policies for gene therapy approval. This inconsistency might impede global cooperation and generate ethical questions about the equitable application of advanced technologies [57]. Emphasizing the need for openness and international cooperation, the World Health Organization has demanded a thorough global framework to guarantee ethical practices in gene editing. This will help to advance public health with new recommendations on human genome editing, aimed at the same goal. These difficulties highlight the need for world cooperation to create universal ethical and legal norms, enabling the realization of the advantages of ocular gene therapy anywhere.

## 7. Gene Therapy Approaches for Ocular Conditions

Targeting the basic genetic causes of ocular diseases instead of only offering symptomatic relief, gene therapy has become a transformational medical tool. Advances in molecular biology and viral vector engineering have made exact gene replacement, gene augmentation, and targeted therapeutic gene expression possible, greatly increasing the treatment options in ophthalmology. With different advantages, limitations, and clinical consequences, the two main approaches in ocular gene therapy are non-viral techniques and viral-vector-mediated delivery [56]. Because of their proven capacity to effectively transfer genetic material into particular cell types, viral vectors are usually preferred in ocular gene therapy. Maintaining therapeutic genes expressed at appropriate levels and over long times depends on this efficient delivery. Among the most often used vectors are lentiviruses, adeno-associated viruses (AAV), and adenoviruses. Every one of these vectors has special qualities that could make them more or less appropriate depending on the targeted disease, the necessary length of expression, and the particular types of cells under treatment [56].

### 7.1. Viral-Vector-Mediated Distribution

#### 7.1.1. Adeno-Associated Virus (AAV)

Small, non-pathogenic, single-stranded DNA viruses known for their safety and capacity to generate continuous gene expression in the retina are called adeno-associated virus (AAV) vectors [51]. With their low immunogenicity, great safety profile, and long-term gene expression, AAV vectors are today the gold standard in ocular gene therapy. AAV vectors provide transgenes episomally, unlike integrating vectors, which greatly lowers risks related to genomic integration, including insertional mutagenesis [58]. Thirteen primate serotypes exist with different capsid compositions that influence their cellular tropism. For example, AAV2 effectively transduces photoreceptors and retinal pigment epithelium (RPE), which forms the basis for Voretigene neparvovec-rzyl (LuxturnaTM), a treatment for RPE65-mediated Leber congenital amaurosis that shows sustained visual improvement [51,59]. Derived from non-human primates, AAV8 shows better photoreceptor transduction following subretinal injection and has a lower seroprevalence of neutralizing antibodies (about 38% compared to almost 70% for AAV8), thus enabling its use in seropositive patients [51,59].

- Genomic capacity: The single-stranded AAV genome size is restricted to roughly 4.7 kb, limiting the delivery of longer coding or regulating sequences. Larger genes, such as *ABCA4* or *USH2A*, are accommodated by dual vector strategies (such as split-intein or overlapping genomes) and minimal promoter designs [58].
- While AAV vectors are usually well tolerated, preexisting anti-AAV antibodies can neutralize vector particles. Intravenous delivery may also cause mild inflammation. Reduced seroreactivity and transient immunosuppression regimens have been developed to improve transduction efficiency and enable re-dosing [51].

#### 7.1.2. Adenovirus

Adenovirus (AdV) vectors are non-enveloped, double-stranded DNA viruses with a packaging capacity of roughly 36 kb that help to deliver either large- or multi-cistronic constructs [60]. Retinoblastoma [61] and models of corneal wound healing have made use of their efficient transduction of both dividing and non-dividing cells. AdV vectors usually cause notable innate immune responses, even if they are quite efficient in ocular cell transduction and are able to carry larger genetic payloads. Although their general acceptance has been limited due to these immunogenic issues, their possible therapeutic importance in oncology uses—including retinoblastoma—remains great [51]. But these drawbacks restrict their use in ophthalmic settings:

- AdV capsids interact with Toll-like receptors (TLR2 and TLR9) on retinal microglia and retinal pigment epithelial (RPE) cells, releasing proinflammatory cytokines (IL-6 and TNF-α) and activating complements [62].
- Acute inflammation: AdV intraocular delivery frequently causes anterior uveitis, vitritis, and macular oedema, which can present as pain, photophobia, and transient vision loss [62,63].
- Transient expression: AdV’s inflammatory response speeds up vector clearance and transduced cell destruction, thus restricting transgene expression to a one-to-four-week range [51].
- High-titer anti-AdV antibodies develop quickly and can impede efficient re-dosing and raise the risk of immune-complex-mediated toxicity [63].
- Higher doses of AdV are linked to spikes in intraocular pressure and anterior chamber inflammation, which calls for careful dosage optimization and maybe prophylactic immunosuppression [63].

Although their complicated manufacturing techniques have hampered their translation into ophthalmic uses, helper-dependent AdV (HD-Ad) vectors—which lack all viral coding sequences—show reduced immunogenicity and prolonged expression in preclinical models [62].

#### 7.1.3. Lentivirus

Complementary DNA is delivered into the host genome by enveloped single-stranded RNA viruses known as lentiviral vectors (LVs), enabling long-term gene expression in both replicating and non-replicating cells [56]. LVs, with a packaging capacity of 8–10 kb, can accommodate therapeutic genes that surpass the limits of AAV, including *ABCA4* (about 6.8 kb) and *USH2A* (about 15.6 kb), fitting for uses in Stargardt disease and Usher syndrome [64]. Modern self-inactivating (SIN) LV designs are meant to exclude viral enhancer/promoter elements to lower the risk of insertional mutagenesis; nevertheless, the residual oncogenic potential calls for careful safety analyses [64].

### 7.2. Non-Viral Gene Distribution Techniques

Although they usually produce reduced immune responses and avoid genomic integration, non-viral systems need other approaches to reach effective transfection [65].

#### 7.2.1. Chemical Controllers

- Liposomes, or lipoplexes: Capturing DNA or RNA, cationic and PEGylated liposomes shield the cargo from nucleases and encourage endocytic absorption. Preclinical ocular studies have shown minimal inflammation and efficient corneal and retinal transduction [65].
- Polymeric nanoparticles (polyplexes): Low-toxicity in vivo biodegradable polymers, including PLGA, chitosan, and PEI, create complexes that allow controlled release and improve cellular absorption [65].
- Dendrimers and peptide vectors: PAMAM dendrimers and cell-penetrating peptides (e.g., TAT) show good safety profiles and help in translocation of nucleic acids across ocular barriers [65].
- Pegylated RNA aptamers: Representing a non-viral anti-angiogenic therapy, pegylated RNA aptamers bind to VEGF with great specificity and can be chemically modified for prolonged intraocular retention [63].

#### 7.2.2. Physical-Based Techniques

To enable gene transfer, physical gene delivery techniques, including electroporation, iontophoresis, and ultrasonic-mediated delivery, momentarily permeabilize ocular cellular barriers. These techniques provide exact spatial and temporal control over gene delivery, extending possible uses over several ocular tissues, including the retina, cornea, and ciliary body [61,65]. The efficacy, safety, and immunological reactions linked with ocular gene therapies are greatly affected by the chosen delivery method. Among the main clinical techniques are intravitreal and subretinal injections.

- Electroporation and iontophoresis: The temporary permeabilization of cell membranes by electrical or ionic currents greatly increases the absorption of naked nucleic acids in corneal and retinal tissues [65].
- Gene gun delivery: Localized corneal transfection is made possible by DNA-coated microparticles being driven into ocular surface cells without clearly damaging any tissue [65].
- Ultrasound-mediated delivery: Targeting specific areas of the posterior segment, focused ultrasonic energy combined with microbubble cavitation improves permeability and endocytic absorption [65].

## 8. Routes of Administration

Therapeutic vectors are delivered by the subretinal injection technique straight into the subretinal space between photoreceptors and retinal pigment epithelial (RPE). The immune-privileged character of this compartment enables great cellular transduction efficiency with much lower immunogenic responses. But this approach calls for advanced surgical knowledge and carries risks, like retinal detachment and transient inflammation; thus, careful patient selection and clinical expertise are necessary [51,60]. By contrast, intravitreal injection offers a minimally invasive substitute that lets vectors be directly injected into the vitreous cavity. Although this method mostly targets the inner retinal layers, it has natural difficulties, including lower vector diffusion through retinal structures and more immune responses. AAV.7m8 and other novel engineered capsids improve transduction efficiency across retinal layers, greatly increasing therapeutic possibilities [51].

- Subretinal injection: Using vectors injected into the subretinal space under the neurosensory retina, this technique achieves high transduction of RPE and photoreceptors in an immune-privileged compartment. The method carries hazards, including detachment, hemorrhage, and transient inflammation, and calls for vitreoretinal expertise [51].
- Intravitreal injection: Targeting inner retinal neurons mostly, this minimally invasive technique, known as intravitreal injection, sends vectors into the vitreous cavity. But local immune responses and diffusion barriers, like the internal limiting membrane, may limit access to the outer retina. Having said that, the engineered capsids, such as AAV7m8, can increase photoreceptor tropism.
- Suprachoroidal delivery: Using vectors between the sclera and choroid, this method—known as suprachoroidal delivery—allows for extensive outer retinal coverage. Though long-term efficacy and safety are still under research, it could lower humoral responses when compared to intravitreal approaches [51].

Although ocular immune privilege exists, gene therapy can cause local inflammation, neutralizing antibody generation, and T-cell-mediated clearance of transduced cells [51]. Low-seroprevalence or engineered capsids, transient immunosuppression, and careful route choice help to reduce antigen presentation (e.g., subretinal rather than intravitreal) [51,62,64].

## 9. Gene Therapy Costs for Eye Diseases

Although it has a significant financial cost, gene therapy offers a transforming solution for IRDs. With peak annual spending reaching roughly USD 25.3 billion and total expenditures exceeding USD 300 billion over 15 years, projections indicate that over 1 million patients might receive gene therapy by 2034 in the United States [66]. Targeting inherited retinal dystrophy brought on by mutations in the RPE65 gene, one of the most well-known treatments is Voretigene neparvovec-rzyl (marketed as Luxturna). Apart from the expenses related to surgical and medical follow-up, Luxturna costs about USD 850,000 for both eyes. This treatment thus causes a great financial load. The manufacturer justifies this price by pointing out possible long-term advantages for consumers, including better quality of life, greater independence, and more workforce participation [67].

Evaluating the cost-effectiveness of gene therapy involves particular difficulties. First, the present evaluation instruments cannot fully reflect how vision loss affects a patient’s quality of life, which results in erroneous estimates of therapeutic efficacy. Second, the natural development of IRDs and the long-term results of this novel treatment are not well known, which influences the validity of cost-effectiveness research [68]. Huygens et al. examined four economic studies done in the United States, the United Kingdom, and the Netherlands to negotiate the methodological complexity in assessing Luxturna’s cost-effectiveness. Their study found that variations in important assumptions—particularly those related to the length of treatment effects, utility estimations, and model structures—resulted in appreciable changes in cost-effectiveness outcomes. Although present economic evaluation models are judged appropriate for gene therapies, the authors underline the need for more explicit methodological guidance to address uncertainties related to long-term outcomes, so as to improve the consistency and dependability of health technology assessments [69].

Using a lifetime health-state model, a recent cost-effectiveness study evaluated Luxturna against conventional therapy. When considering both direct and indirect costs, patients undergoing the gene therapy paid less total lifetime expenses than those undergoing standard treatment and experienced more than nine additional quality-adjusted life years (QALYs). Under several scenarios, the cost-effectiveness stayed favorable, especially when considering long-term society savings from better independence, less carer burden, and better well-being [70].

The great financial cost of gene therapy results from the thorough scientific study and difficult manufacturing techniques required in this kind of treatment. Many patients, meanwhile, find protection from direct costs. Most insurance plans cover a significant amount of the treatment, and manufacturers like Spark Therapeutics offer financial support—including travel assistance and free genetic testing—to help lower access barriers. Ultimately, though, public payers and insurance companies bear the larger financial cost of such treatments, which could affect general healthcare expenditure and premiums [66,67].

By precisely changing the DNA mutations causing hereditary eye diseases, genome editing offers a promising approach for treatment. Four main forms of synthetic nucleases have thus been developed for genome editing: meganucleases, zinc finger nucleases (ZFNs), transcription activator-like effector nucleases (TALENs), and the CRISPR-Cas9 system. Among these, CRISPR-Cas9 is the most preferred, especially for ocular treatments, since its lower cost, simplicity of design, and higher delivery efficiency make sense [48]. Although CRISPR-based genomic treatments have great promise for treating IRDs, their great expenses prevent more general acceptance. In response, a team of analysts is investigating several ways to cut costs. Their projects involve evaluating several kinds of businesses, including socially driven companies and nonprofit drug makers, that put community health above financial gain. Citing California’s low-cost insulin manufacturing program as a model to reduce the financial load of advanced technologies, they also underlined the value of public investment and government-led projects. Using point-of-care models to decentralize the manufacturing process will also help hospitals to create treatments right there at far lower costs. Furthermore, regulatory changes were suggested, mostly aimed at streamlining early-phase clinical trial requirements and helping to reduce development expenses. These approaches, taken together, seek to improve the accessibility and cost of CRISPR-based treatments without sacrificing efficacy or safety [71].

## 10. Conclusions

Gene therapy is transforming the treatment of eye and retinal diseases by addressing their root causes through genetic interventions. Luxturna, an FDA-approved medication, along with a promising new CRISPR-based treatment, offers hope for patients. However, access to these therapies is often limited due to immunological concerns, potential toxicity, and high costs. AAV remains the most widely used vector for delivering such therapies; nevertheless, emerging non-viral options are on the horizon. Enhancing the sensitivity, specificity, and safety of gene-therapy-based medications is essential for improving patient quality of life and achieving long-term vision restoration.

## Figures and Tables

**Table 1:** Ongoing retinal gene therapy clinical trials.

Ocular Disease	Gene Therapy Code	Route of Administration	Study Name	Study Type	Phase	Sponsor	Trial Number
RP	MCO-010	IVI	REMAIN	Observational	-	Nanoscope Therapeutics Inc.	NCT06162585
RP	vMCO-I	IVI	EXTEND	Observational	-	Nanoscope Therapeutics Inc.	NCT05921162
RP	OCU400-301	SRI	liMeliGhT	Interventional	III	Ocugen	NCT06388200
RP	GS030-DP	IVI	PIONEER	Interventional	I/II	GenSight Biologics	NCT03326336
RP	ZM-02	IVI	MOON	Interventional	I	Zhongmou Therapeutics	NCT06292650
RP due to PDE6A	rAAV.hPDE6A	SRI	Pigment	Interventional	I/II	STZ eyetrial	NCT04611503
RP due to PDE6B	AAV2/5-hPDE6B	SRI	-	Interventional	I/II	eyeDNA Therapeutics	NCT03328130
RP/Usher Syndrome Type 2	Ultevursen	IVI	LUNA	Interventional	II	Laboratoires Thea	NCT06627179
RP due to RHO, PDE6A, or PDE6B gene	SPVN06	SRI	PRODYGY	Interventional	I/II	SparingVision	NCT05748873
RP due to CNGA1	VG901	IVI	-	Interventional	I	ViGeneron GmbH	NCT06291935
RP due to RLBP1	CPK850	SRI	-	Interventional	I/II	Novartis Pharmaceuticals	NCT03374657
Autosomal dominant RP due to P23H	QR-1123	IVI	AURORA	Interventional	I/II	ProQR Therapeutics	NCT04123626
XLRP	AAV5 hRKp.RPGR	SRI	-	Interventional	II	Janssen Research & Development, LLC	NCT06646289
XLRP	4D-125	IVI	-	Interventional	I/II	4D Molecular Therapeutics	NCT04517149
XLRP	AGTC-501	SRI	SKYLINE	Interventional	II	Beacon Therapeutics	NCT06333249
XLRP	AGTC-501	SRI	-	Interventional	II/III	Beacon Therapeutics	NCT04850118
XLRP due to RPGR	AAV5-hRKp.RPGR	SRI	-	Observational	-	Janssen Research & Development, LLC	NCT04312672
XLRP due to RPGR	AAV5-hRKp.RPGR	SRI	-	Interventional	III	Janssen Research & Development, LLC	NCT04794101
XLRP due to RPGR	AGTC-501	SRI	DAWN	Interventional	II	Beacon Therapeutics	NCT06275620
XLRP due to RPGR	FT-002	Intraocular injection	-	Interventional	I	Frontera Therapeutics	NCT05874310
XLRP due to RPGR	rAAV2tYF-GRK1-RPGR	IVI	HORIZON	Interventional	I/II	Beacon Therapeutics	NCT03316560
XLRP due to RPGR	FT-002	SRI	-	Interventional	I/II	Frontera Therapeutics	NCT06492850
LCA5	OPGx-001	SRI	LCA5-IRD	Interventional	I/II	Opus Genetics Inc.	NCT05616793
LCA 10	Sepofarsen	IVI	ILLUMINATE	Interventional	II/III	ProQR Therapeutics	NCT03913143
LCA10	Sepofarsen	IVI	HYPERION	Interventional	III	Laboratoires Thea	NCT06891443
LCA 10	EDIT-101	SRI	-	Interventional	I/II	Editas Medicine Inc.	NCT03872479
LCA due to RPE 65	rAAV2-CBSB-hRPE65	SRI	-	Interventional	I	University of Pennsylvania	NCT00481546
LCA due to RPE65	AAV2-hRPE65v2	SRI	-	Interventional	III	Spark Therapeutics Inc.	NCT00999609
LCA due to RPE 65	HG004	NA	STAR	Interventional	I/II	HuidaGene Therapeutics Co., Ltd.	NCT05906953
LCA due to RPE 65	HG004	SRI	LIGHT	Interventional	I	Xinhua Hospital, Shanghai Jiao Tong University School of Medicine	NCT06088992
LCA due to RPE 65	FT-001	SRI	-	Interventional	I/II	Frontera Therapeutics	NCT05858983
LCA due to RPE 65	LX101	SRI	-	Interventional	NA	Shanghai General Hospital, Shanghai Jiao Tong University School of Medicine	NCT06024057
LCA due to RPE65	Voretigene neparvovec	SRI	-	Interventional	III	Novartis Pharmaceuticals	NCT04516369
LCA due to RPE65	LX101	SRI	-	Interventional	I/II	Innostellar Biotherapeutics Co., Ltd	NCT06196827
RDH12 retinopathy	PUMCH-E101	IVI	-	Interventional	I	Peking Union Medical College Hospital	NCT06749639
Stargardt Disease	ACDN-01	SRI	STELLAR	Interventional	I/II	Ascidian Therapeutics Inc.	NCT06467344
Stargardt Disease	MCO-010	IVI	SUSTAIN	Observational	-	Nanoscope Therapeutics Inc.	NCT06048185
Stargardt Disease	JWK006	SRI	-	Interventional	I/II	West China Hospital	NCT06300476
XLRS	ATSN-201	SRI	LIGHTHOUSE	Interventional	I/II	Atsena Therapeutics Inc.	NCT05878860
XLRS	ZM-01	IVI	-	Interventional	I	Zhongmou Therapeutics	NCT06066008
XLRS	JWK002	SRI	-	Interventional	I	West China Hospital	NCT06345898
XLRS	IVB102	IVI	-	Interventional	I	InnoVec Biotherapeutics Inc.	NCT06289452
Achromatopsia	rAAV2tYF-PR1.7-hCNGB3	SRI	A Clarity Clinical Trial	Interventional	I/II	Beacon Therapeutics	NCT02599922
Achromatopsia due to CNGA3	AGTC-402	SRI	A Clarity Clinical Trial	Interventional	I/II	Beacon Therapeutics	NCT02935517
Achromatopsia due to CNGA3	rAAV.hCNGA3	SRI	Colourbridge	Interventional	I/II	STZ eyetrial	NCT02610582
Choroideremia	4D-110	IVI	-	Interventional	I	4D Molecular Therapeutics	NCT04483440
RP, Choroideremia	RTx-015	IVI	ENVISION	Interventional	I	Ray Therapeutics Inc.	NCT06460844
Choroideremia XLRP	BIIB111 BIIB112	SRI SRI	SOLSTICE	Interventional	III	NightstaRx Ltd., a Biogen Company	NCT03584165
Bietti Crystalline Corneoretinal Dystrophy	NGGT001	SRI	-	Interventional	I/II	NGGT (Suzhou) Biotechnology Co., Ltd.	NCT06706427
Bietti’s Crystalline Dystrophy	ZVS101e	SRI	-	Interventional	III	Chigenovo Co., Ltd.	NCT06743646
Bietti’s Crystalline Dystrophy	VGR-R01	SRI	-	Interventional	III	Shanghai Vitalgen BioPharma Co., Ltd.	NCT06699108
nAMD	NG101 AAV	SRI	-	Interventional	I/II	Neuracle Genetics Inc.	NCT05984927
nAMD	FT-003	Intraocular Injection	-	Interventional	I	Frontera Therapeutics	NCT05611424
nAMD	FT-003	Intraocular injection	-	Interventional	I/II	Frontera Therapeutics	NCT06492863
nAMD	LX102	SRI	-	Interventional	I	Innostellar Biotherapeutics Co., Ltd	NCT06198413
nAMD	SKG0106	IVI	-	Interventional	I	Youxin Chen	NCT06213038
nAMD	SKG0106	IVI	-	Interventional	I/II	Skyline Therapeutics (US) Inc.	NCT05986864
nAMD	KH631	Intraocular injection	-	Interventional	I	Chengdu Origen Biotechnology Co., Ltd.	NCT05657301
nAMD	KH631	SRI	-	Interventional	I/II	Chengdu Origen Biotechnology Co., Ltd.	NCT05672121
nAMD	ADVM-022	IVI	OPTIC-EXT	Observational	-	Adverum Biotechnologies Inc.	NCT04645212
nAMD	KH658	SCSI	-	Interventional	I/II	Chengdu Origen Biotechnology Co., Ltd.	NCT06458595
nAMD	RGX-314	SCSI	AAVIATE	Interventional	II	AbbVie	NCT04514653
nAMD	RGX-314	SRI	RGX-314 SRLTFU	Interventional	II	AbbVie	NCT03999801
nAMD	RGX-314	SRI	ASCENT	Interventional	III	AbbVie	NCT05407636
nAMD	LX102	SRI	VENUS	Interventional	II	Innostellar Biotherapeutics Co., Ltd.	NCT06196840
nAMD	LX102	SRI	-	Interventional	I	Innostellar Biotherapeutics Co., Ltd.	NCT06198413
nAMD	ABBV-RGX-314	SRI	ATMOSPHERE	Interventional	II/III	AbbVie	NCT04704921
nAMD	RRG001	SRI	-	Interventional	I/II	Shanghai Refreshgene Technology Co., Ltd.	NCT06141460
nAMD	4D-150	IVI	-	Interventional	I/II	4D Molecular Therapeutics	NCT05197270
nAMD	EXG202	IVI	-	Interventional	I	Hangzhou Jiayin Biotech, Ltd.	NCT06888492
nAMD	HG202 CRISPR-Cas13 RNA-editing	SRI	SIGHT-I	Interventional	I	HuidaGene Therapeutics Co., Ltd.	NCT06031727
nAMD	KH658	SCSI	-	Interventional	I	Chengdu Origen Biotechnology Co., Ltd.	NCT06825858
nAMD	EXG102-031	SRI	Everest	Interventional	I	Exegenesis Bio	NCT05903794
nAMD	EXG102-031	SRI	Everest LTFU	Interventional	I	Exegenesis Bio	NCT06817343
nAMD	HG202 CRISPR-Cas13 (hfCas13Y)	SRI	BRIGHT	Interventional	I	HuidaGene Therapeutics Co., Ltd.	NCT06623279
GA due to AMD	GT005	NA	ORACLE	Interventional	II	Gyroscope Therapeutics Limited	NCT05481827
nAMD	4D-150	IVI	-	Interventional	III	4D Molecular Therapeutics	NCT06864988
nAMD	ADVM-022	IVI	LUNA	Interventional	II	Adverum Biotechnologies Inc.	NCT05536973
Dry AMD	Elamipretide	SC	ReNEW	Interventional	III	Stealth BioTherapeutics Inc.	NCT06373731
nAMD	Ixo-vec	IVI	ARTEMIS	Interventional	III	Adverum Biotechnologies Inc.	NCT06856577
DME	FT-003	Intraocular injection	-	Interventional	I/II	Frontera Therapeutics	NCT06492876
DME	FT-003	Intraocular injection	-	Interventional	I	Frontera Therapeutics	NCT05916391
DME	4D-150	IVI	-	Interventional	II	4D Molecular Therapeutics	NCT05930561
DME	SKG0106	IVI	-	Interventional	I	Wang Min	NCT06237777
DR without CI-DME	RGX-314	SCSI	ALTITUDE^®^	Interventional	I	AbbVie	NCT04567550
DME	ADVM-022	IVI	INFINITY-EXT	Observational	-	Adverum Biotechnologies Inc.	NCT05607810

Only active studies included (recruiting, non-recruiting, and enrolling by invitation). Terminated, completed, withdrawn, suspended, or unknown status studies were not included. Source: https://www.clinicaltrials.gov. Ocular disease abbreviations and gene therapy code descriptions are provided in the final section of the manuscript after the Conclusion.

## Abbreviations

RP	Retinitis pigmentosa
XLRP	X-linked retinitis pigmentosa
LCA	Leber congenital amaurosis
XLRS	X-linked retinoschisis
nAMD	Neovascular age-related macular degeneration
GA	Geographic atrophy
DME	Diabetic macular edema
CI-DME	Central involving diabetic macular edema
DR	Diabetic retinopathy
IVI	Intravitreal injection
SRI	Subretinal injection
SCSI	Suprachoroidal space injection
**Gene Therapy Code Descriptions**	
MCO-010	Optogenetic therapy using multi-characteristic opsin (Nanoscope Therapeutics)
vMCO-I	Variant of MCO-010; optogenetic therapy (Nanoscope Therapeutics)
OCU400-301	NR2E3-based gene modifier therapy for IRDs (Ocugen)
GS030-DP	Optogenetic therapy plus stimulation device (GenSight Biologics)
ZM-02	AAV-based therapy (Zam Therapeutics)
rAAV.hPDE6A	AAV therapy delivering PDE6A gene for RP
AAV2/5-hPDE6B	AAV2/5 vector delivering PDE6B gene for RP
Ultevursen	Antisense oligonucleotide targeting USH2A (ProQR)
SPVN06	Neuroprotective AAV gene therapy (SparingVision)
VG901	Investigational gene therapy (Vico Therapeutics)
CPK850	AAV8-based gene therapy for RPE65 mutations (Novartis)
QR-1123	Antisense oligo targeting RHO-P23H mutation (ProQR)
AAV5 hRKp.RPGR	AAV5 vector with RPGR gene under RK promoter
4D-125	RPGR-targeting gene therapy by 4D Molecular Therapeutics
AGTC-501	AAV-based gene therapy for RPGR mutations (AGTC)
FT-002	F-star Therapeutics pipeline candidate; retinal focus
rAAV2tYF-GRK1-RPGR	Modified AAV2 vector for RPGR delivery under GRK1 promoter
OPGx-001	AAV gene therapy targeting GUCY2D (Opus Genetics)
Sepofarsen	Antisense oligonucleotide targeting CEP290 (ProQR)
EDIT-101	CRISPR/Cas9 genome editing for CEP290 (Editas Medicine)
rAAV2-CBSB-hRPE65	AAV2 vector delivering RPE65 under synthetic promoter
AAV2-hRPE65v2	Optimized RPE65 gene therapy (Luxturna)
HG004	Gene therapy from HuidaGene Therapeutics
FT-001	F-star Therapeutics candidate
LX101	AAV-based gene therapy for AMD/IRD (Luxna Biotech)
Voretigene neparvovec	FDA-approved RPE65 gene therapy (Luxturna)
PUMCH-E101	Gene editing therapy from PUMCH
ACDN-01	AavantiBio-associated candidate; RPGR-related
JWK006	JW Therapeutics gene therapy
ATSN-201	Atsena Therapeutics gene therapy for MYO7A (USH1B)
ZM-01	Zam Therapeutics pipeline candidate
JWK002	JW Therapeutics pipeline therapy
IVB102	Investigational therapy by Iveric Bio
rAAV2tYF-PR1.7-hCNGB3	AAV2 variant for CNGB3 delivery for achromatopsia
AGTC-402	Gene therapy for CNGB3-associated achromatopsia
rAAV.hCNGA3	AAV therapy delivering CNGA3 for achromatopsia
4D-110	AAV-based therapy by 4DMT for RPE65
RTx-015	Optogenetic therapy by Ray Therapeutics
BIIB111	Biogen gene therapy for choroideremia (CHM)
BIIB112	Biogen gene therapy for CNGB3 (achromatopsia)
NGGT001	NeuroGene gene therapy
ZVS101e	Zvesda Therapeutics pipeline candidate
VGR-R01	Gene therapy candidate from Vigeneron
NG101 AAV	NeuroGene investigational AAV therapy
FT-003	F-star Therapeutics gene therapy candidate
LX102	Gene therapy from Luxna Biotech
SKG0106	Gene therapy from Syngene Korea
ADVM-022	AAV.7m8-aflibercept gene therapy for wet AMD (Adverum)
KH658	KH Life Sciences gene therapy
RGX-314	AAV8 vector expressing anti-VEGF Fab (Regenxbio)
ABBV-RGX-314	RGX-314 program in partnership with AbbVie
RRG001	Gene therapy from Ray Therapeutics or Regenxbio
4D-150	Dual transgene AAV for VEGF-A and VEGFR1 (4DMT)
EXG202	Exegenesis Bio gene therapy candidate
HG202	HuidaGene RNA-targeted gene therapy
CRISPR-Cas13 RNA-editing	RNA-targeted editing therapy using Cas13 enzyme
EXG102-031	Exegenesis gene therapy for retinal degeneration
CRISPR-Cas13 (hfCas13Y)	High-fidelity Cas13 variant for RNA editing
GT005	AAV therapy for dry AMD targeting complement factor I
Elamipretide	Mitochondria-targeted peptide for AMD (Stealth BioTherapeutics)
Ixo-vec	AAV8 vector expressing aflibercept (Iveric Bio)
